# Supplementary material for: Vitamin D Modified DSS-Induced Colitis in Mice via STING Signaling Pathway
Source: Biology (Basel). 2025 Jun 18;14(6):715. doi: 10.3390/biology14060715 (PMC12190092; doi:10.3390/biology14060715)
Supplement: Supplementary file 1 [file biology-14-00715-s001.zip › Supplementary Materials S1: 16s-report of gut microbiota/Groups/Lefse_Analysis/biomarkers_raw_images/1_d__Bacteria-p__Actinobacteriota-c__Coriobacteriia-o__Coriobacteriales-f__Atopobiaceae-g__Coriobacteriaceae_UCG_002.pdf]

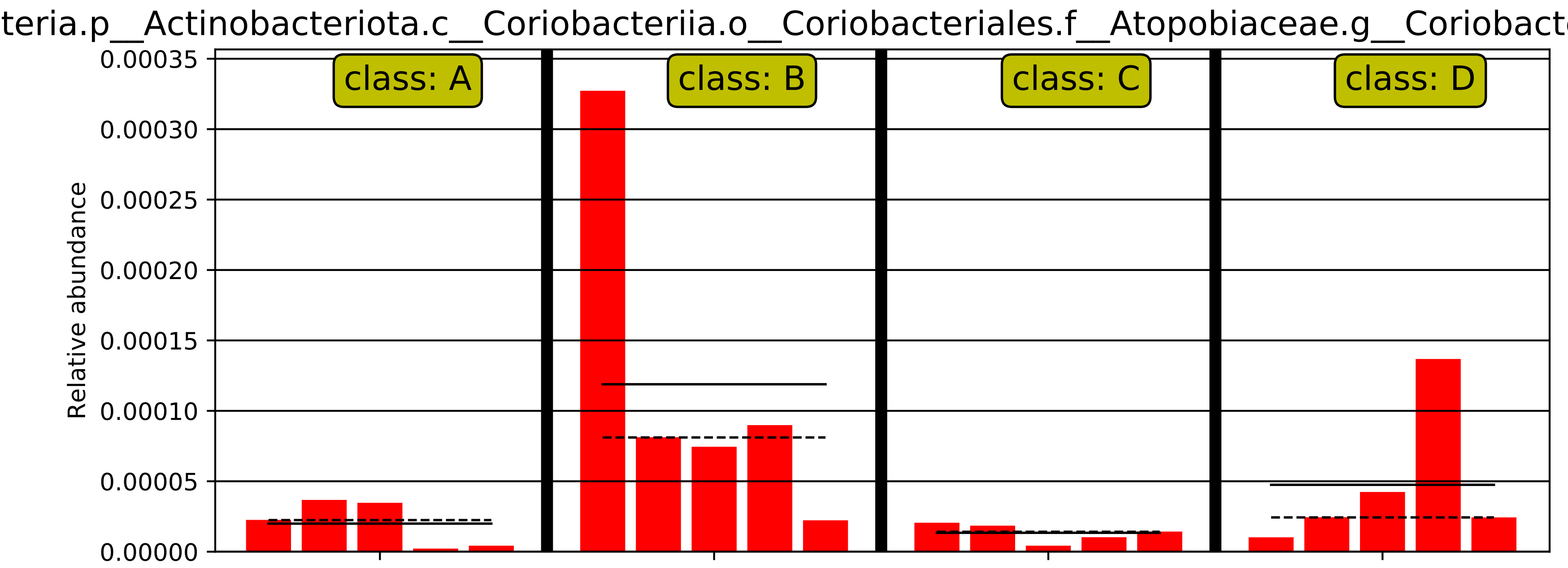
teria.p__Actinobacteriota.c__Coriobacteriia.o__Coriobacteriales.f__Atopobiaceae.g__Coriobacteriaceae_
Relative abundance
0.00035
0.00030
0.00025
0.00020
0.00015
0.00010
0.00005
0.00000
class: A
class: B
class: C
class: D
